# Porcine Epidemic Diarrhea Virus Infection Disrupts the Nasal Endothelial Barrier To Favor Viral Dissemination

**DOI:** 10.1128/jvi.00380-22

**Published:** 2022-04-18

**Authors:** Jianda Li, Yuchen Li, Peng Liu, Xiuyu Wang, Yichao Ma, Qiu Zhong, Qian Yang

**Affiliations:** a MOE Joint International Research Laboratory of Animal Health and Food Safety, College of Veterinary Medicine, Nanjing Agricultural University, Nanjing, Jiangsu, People’s Republic of China; Instituto de Biotecnologia/UNAM

**Keywords:** PEDV, endothelial dysfunction, MMP-7, tight junctions, ICAM-1, transendothelial migration

## Abstract

Crossing the endothelium from the entry site and spreading in the bloodstream are crucial but obscure steps in the pathogenesis of many emerging viruses. Previous studies confirmed that porcine epidemic diarrhea virus (PEDV) caused intestinal infection by intranasal inoculation. However, the role of the nasal endothelial barrier in PEDV translocation remains unclear. Here, we demonstrated that PEDV infection causes nasal endothelial dysfunction to favor viral dissemination. Intranasal inoculation with PEDV compromised the integrity of endothelial cells (ECs) in nasal microvessels. The matrix metalloproteinase 7 (MMP-7) released from the PEDV-infected nasal epithelial cells (NECs) contributed to the destruction of endothelial integrity by degrading the tight junctions, rather than direct PEDV infection. Moreover, the proinflammatory cytokines released from PEDV-infected NECs activated ECs to upregulate ICAM-1 expression, which favored peripheral blood mononuclear cells (PBMCs) migration. PEDV could further exploit migrated cells to favor viral dissemination. Together, our results reveal the mechanism by which PEDV manipulates the endothelial dysfunction to favor viral dissemination and provide novel insights into how coronavirus interacts with the endothelium.

**IMPORTANCE** The endothelial barrier is the last but vital defense against systemic viral transmission. Porcine epidemic diarrhea virus (PEDV) can cause severe atrophic enteritis and acute viremia. However, the mechanisms by which the virus crosses the endothelial barrier and causes viremia are poorly understood. In this study, we revealed the mechanisms of endothelial dysfunction in PEDV infection. The viral infection activates NECs and causes the upregulation of MMP-7 and proinflammatory cytokines. Using NECs, ECs, and PBMCs as *in vitro* models, we determined that the released MMP-7 contributed to the destruction of endothelial barrier, and the released proinflammatory cytokines activated ECs to facilitate PBMCs migration. Moreover, the virus further exploited the migrated cells to promote viral dissemination. Thus, our results provide new insights into the mechanisms underlying endothelial dysfunction induced by coronavirus infection.

## INTRODUCTION

The nasal mucosa is a critical component of the mucosal immunity in the upper respiratory tract. It plays an important role in host defense and immune homeostasis between the commensal microbiota and invading pathogens. Therefore, as the first site directly exposed to inhaled pathogens, the nasal epithelium is exploited by various viruses for viral entry, such as severe acute respiratory syndrome coronavirus 2 (SARS-CoV-2), influenza virus, and respiratory syncytial virus (1–3). Like SARS-CoV-2, another enteric infectious coronavirus, porcine epidemic diarrhea virus (PEDV), exhibits tropism to nasal epithelial cells (NECs) and can cause diarrhea and dehydration in suckling piglets through nasal infection (4, 5). However, the mechanism by which the virus enters the bloodstream from the nasal epithelium remains unclear.

Abundant microvessels exist under the nasal epithelium, forming the essential endothelial barrier that prevents pathogens from entering the blood. Endothelial barrier homeostasis is maintained by tight junctions (TJs) and adherens junctions (AJs). Endothelial cells (ECs) are central orchestrators of vascular permeability, leukocyte recruitment, and tissue damage. Moreover, ECs maintain tight control of pathogens and inflammatory signals, establishing the innate immune response to infection (6, 7). Many emerging viruses, including henipavirus, hantavirus, and SARS-CoV-2, show endothelial tropism, disrupting endothelial barriers via viral infection (8–10). Severe viral infection more often than not includes signs related to disruption of the endothelial barrier in the absence of endothelial infection, resulting from immune signals from infected cells and recruitment of immune cells (11, 12). Whether through direct or indirect viral infection, the virus disrupts the endothelial barrier by targeting ECs, facilitating viral transmission in the body.

PEDV, a member of the *Coronaviridae*, causes considerable economic losses to the global swine industry. PEDV primarily infects small intestinal epithelia, causing villous necrosis and atrophy. In addition to the fecal-oral route, the fecal-nasal route is another route of PEDV transmission through airborne aerosolized PEDV particles (4, 5). Infectious PEDV presents in serum or blood cells in piglets infected with PEDV (13, 14), suggesting that the virus possesses the ability to cross the endothelial barrier and cause viremia.

The ability of the virus to disrupt the endothelial barrier allows the dissemination of infection and facilitates access to specific tissues. In the present study, the hypothesis that PEDV infection disrupts the nasal endothelial barrier to favor its dissemination is proposed. Neonatal piglets were challenged with PEDV intranasally to verify our hypothesis. NECs, ECs, and peripheral blood mononuclear cells (PBMCs) were used as *in vitro* models to reveal the mechanism of endothelial dysfunction induced by PEDV infection. This study is the first to demonstrate that PEDV infection disrupts the endothelial barrier, providing new insights into the role of endothelial dysfunction in coronavirus infection.

## RESULTS

### Intranasal inoculation with PEDV impairs the integrity of nasal microvessels.

**FIG 1:**
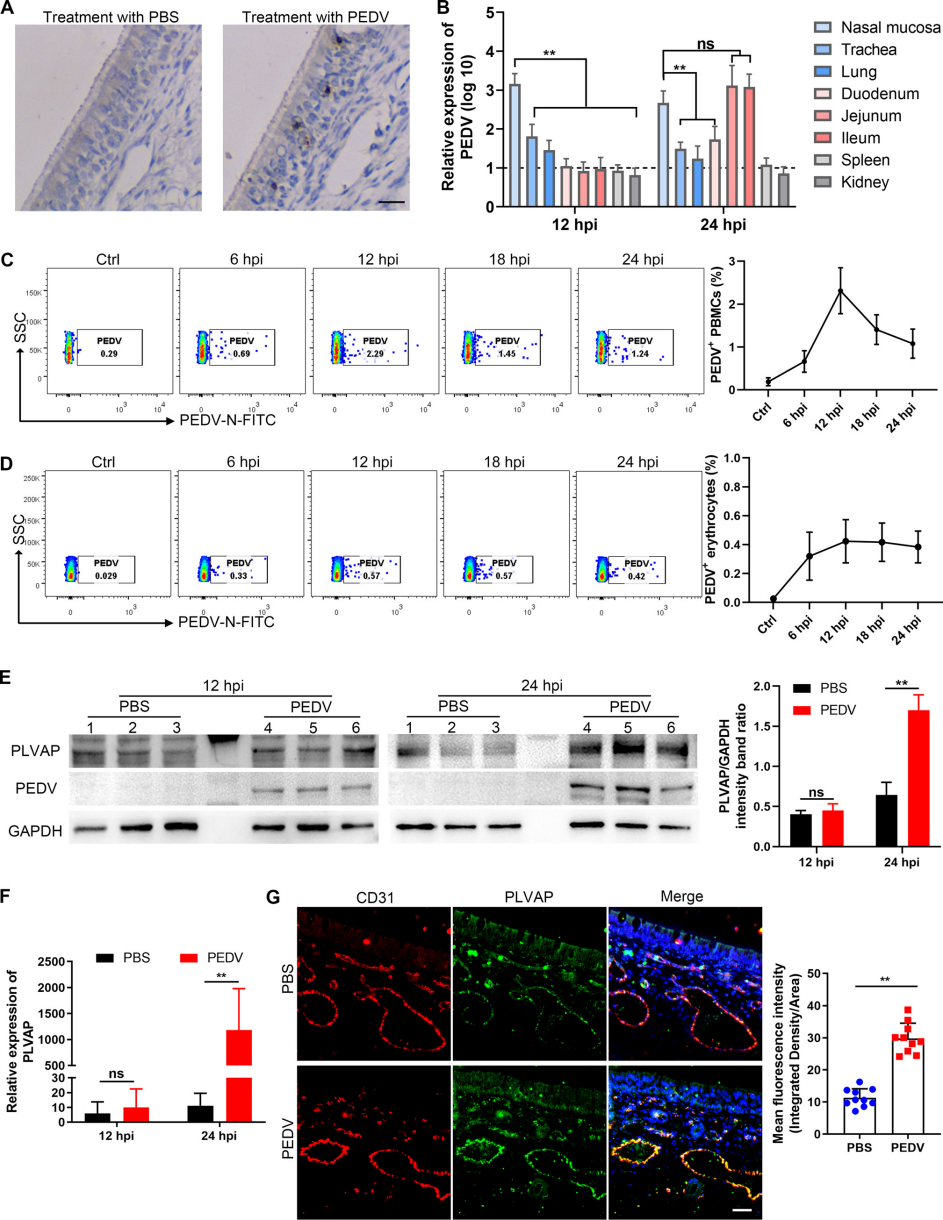
Intranasal inoculation with PEDV impairs the integrity of nasal microvessels. Neonatal piglets were challenged with PEDV via intranasal inoculation. (A) Distribution of PEDV in nasal mucosa at 24 hpi, determined by IHC staining. Bars, 20 μm. (B) Viral RNA in various tissues, detected at 12 hpi and 24 hpi. (C and D) PBMCs and erythrocytes were isolated from neonatal-piglet blood at the indicated times. The percent PEDV^+^ PBMCs (C) and PEDV^+^ erythrocytes (D) was determined by flow cytometric analysis. (E and F) The levels of PEDV-N protein and PLVAP in nasal mucosa were detected at 12 hpi and 24 hpi by Western blotting (E) and RT-qPCR (F). (G) IFA analysis assessed PLVAP expression in the endothelium (CD31) of the nasal mucosa following PEDV intranasal inoculation. Blue, DAPI; red, CD31; green, PLVAP. Bars, 25 μm. The mean fluorescence intensity (MFI) of PLVAP was evaluated by using Image J. Data are means and SD from three independent experiments. **, *P < *0.01; ns, not significant.

Neonatal piglets were challenged with PEDV through nasal spraying. The virus-positive cells were mainly distributed in the nasal epithelium, as determined by immunohistochemistry (IHC) analysis at 24 h postinoculation (hpi) (Fig. 1A). At 12 hpi, the virus was detected in the nasal mucosa, trachea, and lung, while it was undetected in intestinal and other tissues (Fig. 1B). At 24 hpi, viral RNA expression occurred in different tissues, and higher levels of viral RNA were detected in the jejunum and ileum than in other tissues (Fig. 1B). We further isolated PBMCs and erythrocytes from neonatal piglet blood at the indicated times to detect PEDV. The flow cytometry analyses showed that PEDV was presented in PBMCs at a lower percentage at 6 hpi, reached a peak at 12 hpi, and gradually decreased (Fig. 1C). Conversely, PEDV levels in erythrocytes were continuously low from 6 hpi to 24 hpi (Fig. 1D). These results suggest that PEDV may possess the ability to cross the endothelium and enter the bloodstream after intranasal inoculation. To examine whether PEDV intranasal infection affects endothelial integrity, we analyzed the expression of plasmalemma vesicle-associated protein (PLVAP), a marker of ECs’ permeability (15). The PLVAP level in nasal microvessels was increased following intranasal infection with PEDV but was unaffected in the early stage of PEDV infection (Fig. 1E and F). Moreover, immunofluorescence analysis (IFA) revealed that PEDV infection elevated PLVAP expression in nasal microvessels at 24 hpi (Fig. 1G). These data indicate that PEDV infection indeed increases the permeability of nasal microvessels and impairs the endothelial integrity.

### PEDV has no tropism to ECs.

**FIG 2:**
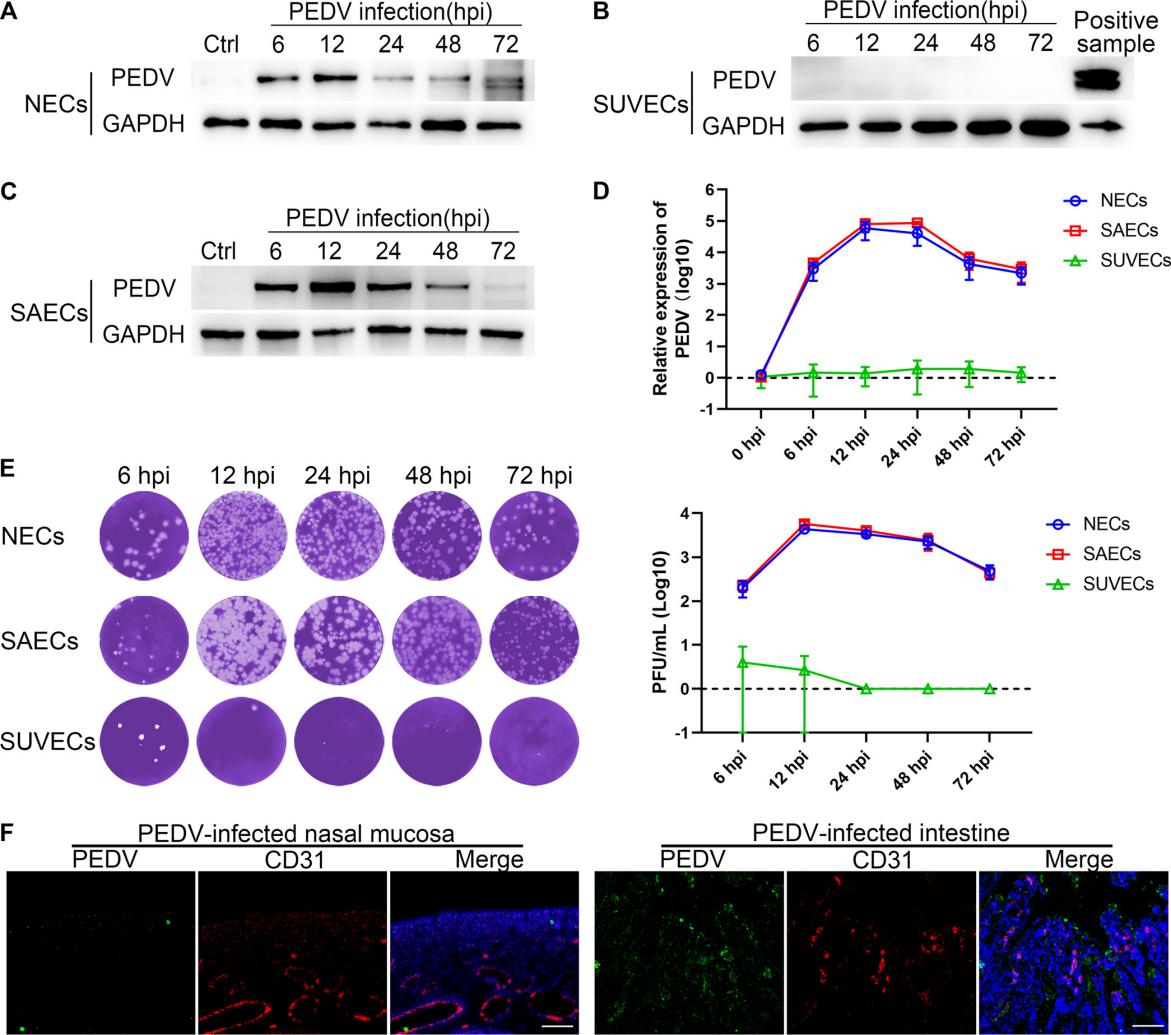
PEDV has no tropism to ECs. NECs, SUVECs, and SAECs were infected with PEDV (MOI, 1). (A to C) The level of PEDV-N protein was assessed in NECs (A), SUVECs (B), and SAECs (C) by Western blotting. (D) The viral RNA levels in NECs, SUVECs, and SAECs were detected by RT-qPCR. (E) The yields of the progeny virus in supernatants of PEDV-infected NECs, SUVECs, and SAECs were measured using plaque assays. (F) Sections of PEDV-infected nasal mucosa and PEDV-infected intestine were observed by IFA. No colocalization between PEDV and CD31 was observed. Blue, DAPI; red, CD31; green, PEDV. Bars, 50 μm. Data are means and SD from three independent experiments.

NECs were isolated from the nasal mucosa of neonatal piglets. A swine umbilical vein cell (SUVEC) line was used as an EC model *in vitro*. To explore the tropism of PEDV, both NECs and SUVECs were infected with PEDV at a multiplicity of infection (MOI) of 1. Following PEDV infection, the level of PEDV N protein in NECs peaked at 12 hpi and gradually decreased (Fig. 2A and D). Although viral RNA decreased at 24 hpi, the progeny virus was still released during PEDV infection until 72 hpi (Fig. 2E). However, no immunoreactivity to viral protein was observed in SUVECs (Fig. 2B). In addition, no viral RNA or progeny virus was detected in SUVECs after PEDV infection (Fig. 2D and E). Furthermore, neither the PEDV-infected nasal mucosa nor the PEDV-infected intestine detected immunoreactivity to viral proteins in the endothelium (Fig. 2F), indicating that PEDV has no tropism to ECs. To further understand how PEDV disrupts the endothelial barrier, a swine airway epithelial cell (SAEC) line was cultured as a nasal epithelium model instead of NECs due to their poor transfection efficiency. Indeed, the trend of viral propagation and release in SAECs was consistent with that in NECs (Fig. 2C to E). Moreover, nasal and airway epithelial cells showed negligible differences in morphology and immune response (16–18).

### Molecules released by PEDV-infected NECs disrupt endothelial barrier integrity.

**FIG 3:**
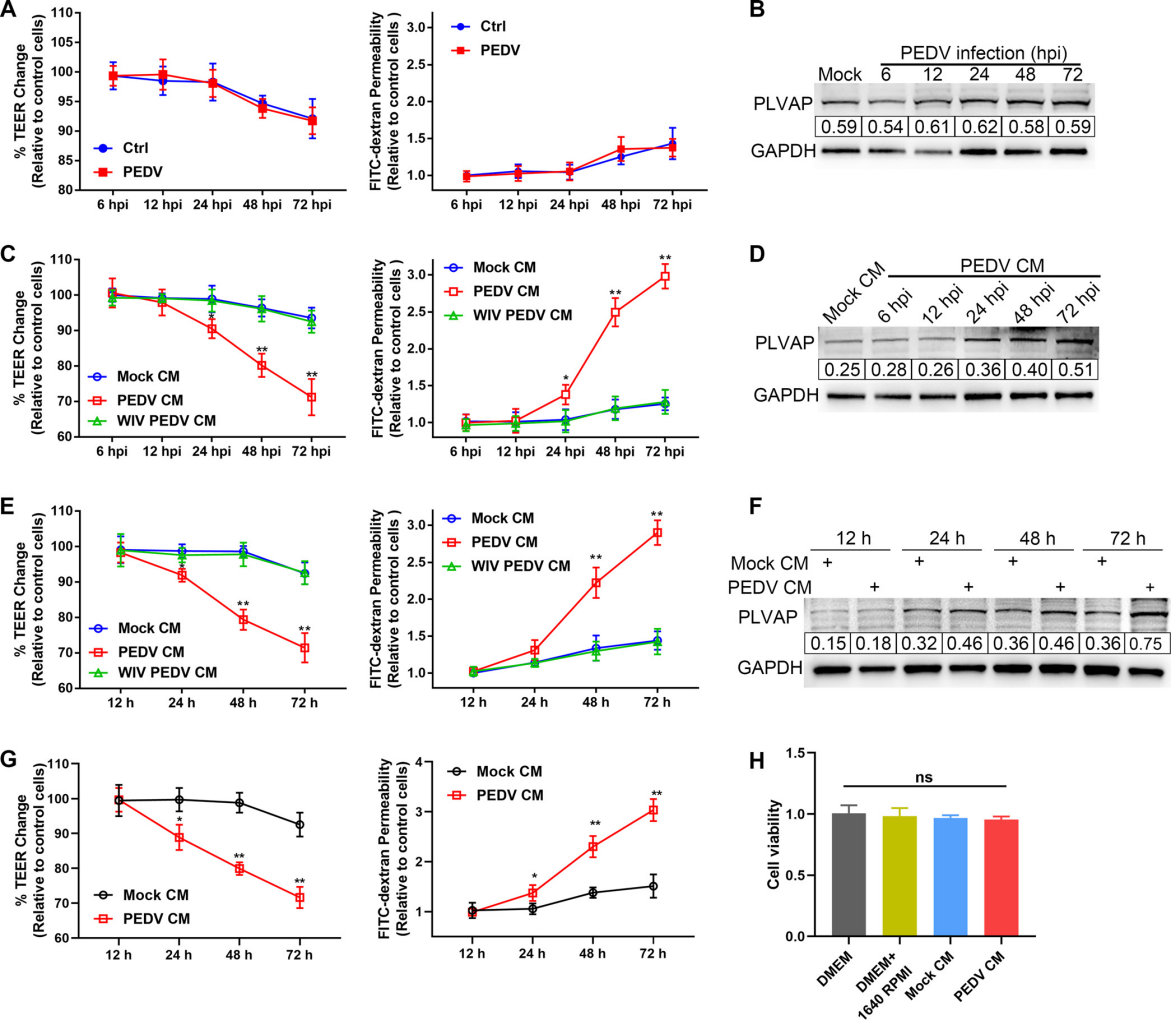
Effects of PEDV infection on the integrity of endothelial barrier. SUVECs were grown on 24-well Transwell inserts until confluence. (A and B) SUVECs were directly inoculated with PEDV (MOI, 1). The TEER and transendothelial permeability were monitored (A). The PLVAP levels in SUVECs were determined by Western blotting (B). (C and D) After infection of SAECs with PEDV or WIV PEDV, the supernatants were collected at the indicated times and mixed with an equal volume of fresh medium. SUVECs were incubated with the manipulated media for 48 h to quantify the TEER and transendothelial permeability (C) and to detect PLVAP levels (D). (E and F) The supernatants were collected from PEDV-infected SAECs or WIV PEDV-infected SAECs at 48 hpi and mixed with an equal volume of fresh medium. SUVECs were incubated with the manipulated medium to monitor the TEER, transendothelial permeability (E), and PLVAP levels (F) at the indicated times. (G) After infection of NECs with PEDV, the supernatants were collected at 48 hpi and mixed with an equal volume of fresh medium. SUVECs were incubated with the manipulated medium for the indicated times to measure the TEER and transendothelial permeability. (H) SUVECs were incubated with different manipulated medium for 48 h. The cell viability of SUVECs was measured by CCK-8 test. Data are means and SD from three independent experiments. *, *P < *0.05; **, *P < *0.01; ns, not significant.

SUVECs monolayers were grown on Transwell filter inserts and then inoculated with PEDV (MOI, 1). Compared to control cells, PEDV-inoculated SUVECs showed constant transendothelial electrical resistance (TEER), transendothelial permeability, and PLVAP levels (Fig. 3A and B). Thus, a hypothesis was proposed that PEDV compromises the endothelial barrier by potential soluble factors released by PEDV-infected cells. SAECs and NECs were infected with PEDV or whole inactivated (WIV) PEDV to verify the hypothesis. Supernatants from mock-, PEDV-, or WIV PEDV-infected cells were collected at different times (6 to 72 hpi) and mixed with an equal volume of fresh medium. These mixtures were designated mock-conditioned medium (mock CM), PEDV-conditioned medium (PEDV CM), and WIV PEDV-conditioned medium (WIV PEDV CM). SUVEC monolayers were incubated with these mixed media for 48 h. In contrast to mock CM and WIV PEDV CM incubation, PEDV CM (collected at 24 to 72 hpi) incubation reduced the TEER and increased the cell permeability (Fig. 3C). Consistent with these results, PLVAP expression in PEDV CM-incubated SUVECs was elevated (Fig. 3D). Moreover, PEDV CM (collected at 48 hpi) increasingly compromised the endothelial barrier over time, as determined by detecting the TEER, transendothelial permeability, and PLVAP levels (Fig. 3E and F). Similarly, the supernatant collected from PEDV-infected NECs decreased the TEER and increased the permeability of SUVECs (Fig. 3G). In contrast, there was no significant difference in cell viability after incubation with mock CM and PEDV CM (Fig. 3H).

### Molecules released by PEDV-infected NECs degrade endothelial TJs.

**FIG 4:**
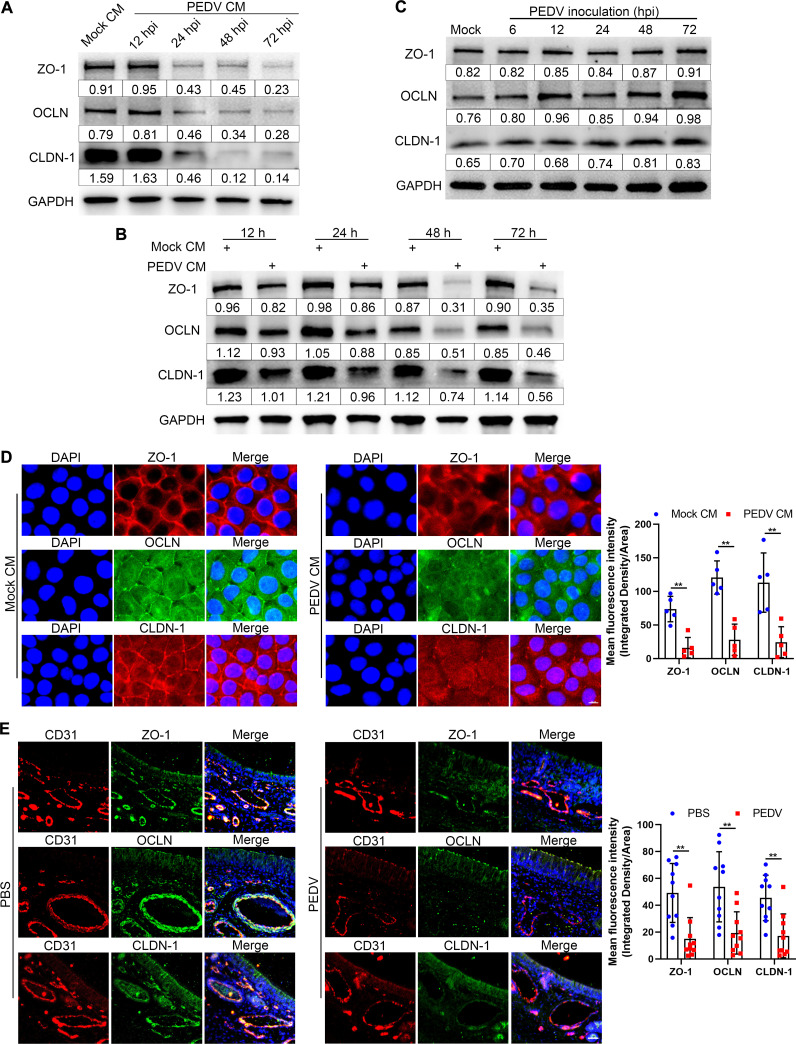
Molecules released by PEDV-infected NECs degrade the endothelial TJs. (A) SUVECs were incubated with PEDV CM (collected at 12 to 72 hpi) for 48 h. The levels of ZO-1, OCLN, and CLDN-1 in SUVECs were detected by Western blotting. (B) SUVECs were incubated with mock or PEDV CM (collected at 48 hpi). The levels of ZO-1, OCLN, and CLDN-1 in SUVECs were assessed at the indicated times by Western blotting. (C) After inoculation of SUVECs with PEDV, the expressions of ZO-1, OCLN, and CLDN-1 were determined at the indicated times by Western blotting. (D) SUVECs were incubated with mock or PEDV CM for 48 h. The degradations of ZO-1 (red), OCLN (green), and CLDN-1 (red) were visualized by IFA staining. Bars, 10 μm. The MFI of ZO-1, OCLN, and CLDN-1 was evaluated using ImageJ. (E) Following intranasal inoculation of piglets with PEDV, the expression levels of ZO-1, OCLN, and CLDN-1 in nasal microvessels were visualized at 24 hpi by IFA staining. Blue, DAPI; red, CD31; green, ZO-1, OCLN, and CLDN-1. Bars, 25 μm. The MFI of ZO-1, OCLN, and CLDN-1 was evaluated by using ImageJ. Data are means and SD from three independent experiments. **, *P < *0.01.

The permeability and integrity of the endothelial barrier are mainly controlled by TJs (19). Thus, the expression of endothelial TJs was determined after incubation of SUVECs with PEDV CM (collected at 12 to 72 hpi) for 48 h. In comparison with mock CM, the PEDV CM collected at 24 to 72 hpi reduced the expression of zonula occludens-1 (ZO-1), occludin (OCLN), and claudin-1 (CLDN-1) (Fig. 4A). In addition, the degradation of endothelial TJs commenced 24 h after incubation with PEDV CM collected at 48 hpi (Fig. 4B). The IFA results also revealed that PEDV CM degraded the expressions of ZO-1, OCLN, and CLDN-1 (Fig. 4D). However, direct inoculation with PEDV did not affect the degradation of endothelial TJs (Fig. 4C). The expression of ZO-1, OCLN, and CLDN-1 was also determined in nasal microvessels to validate whether PEDV infection disrupts the integrity of nasal microvessels. Consistent with *in vitro* results, PEDV intranasal infection resulted in considerable degradation of ZO-1, OCLN, and CLDN-1 in nasal microvessels (Fig. 4E). These data indicate that PEDV exerts a destructive effect on the endothelial barrier through certain soluble factors from PEDV-infected NECs, rather than through a direct role of the virus.

### Molecules released by PEDV-infected NECs impede endothelial barrier repair.

**FIG 5:**
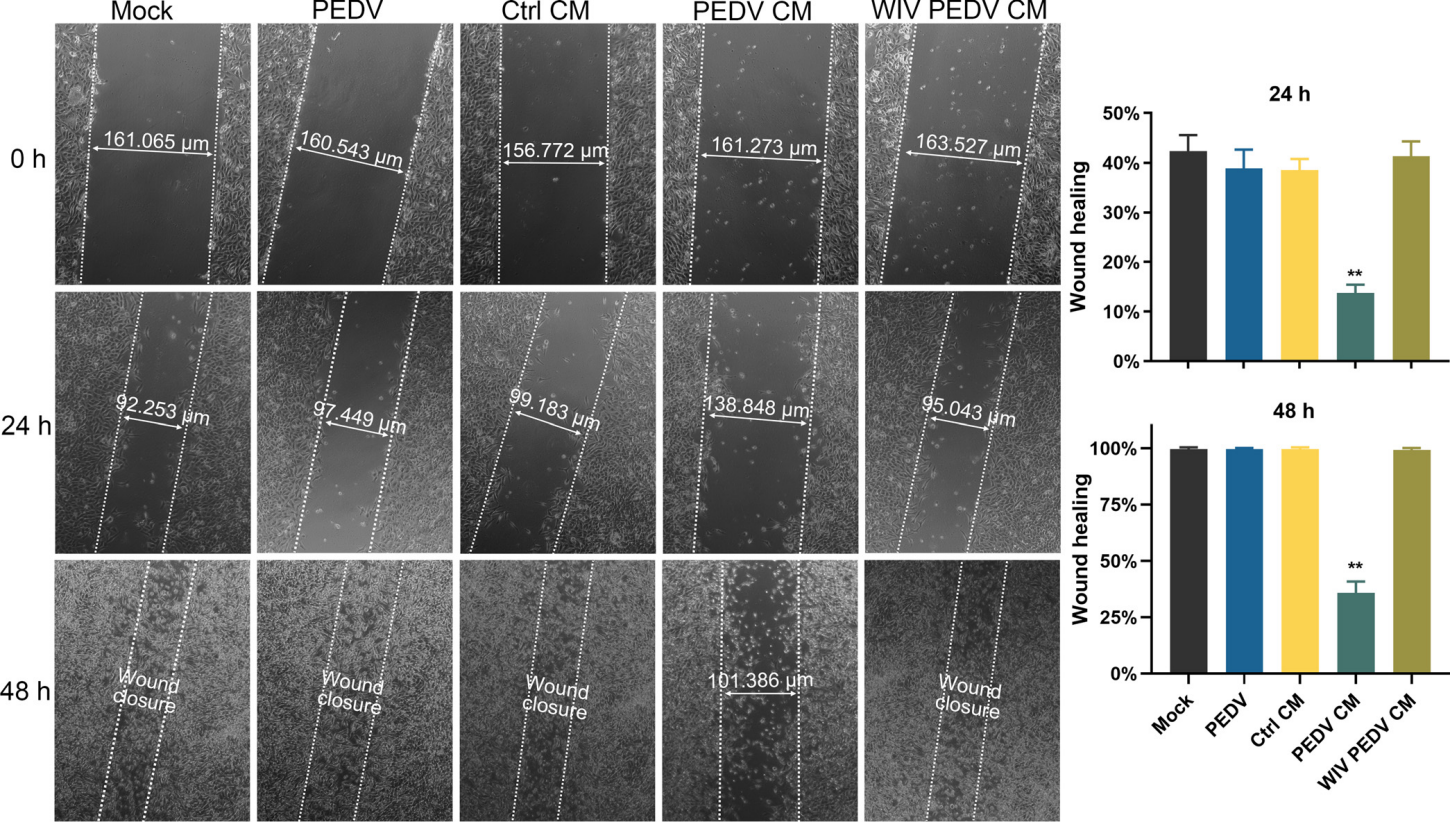
Molecules released by PEDV-infected NECs impede the endothelial barrier repair. After scratching, confluent SUVECs were inoculated with PEDV and incubated with mock, PEDV, or WIV PEDV CM. The wounds were measured using ZEN software. Quantification of wound recovery at 24 hpi and 48 hpi. Data are means and SD from three independent experiments. **, *P < *0.01.

To analyze whether PEDV CM affects the repair of the endothelial barrier, we measured the level of wound healing using the scratch assay. As shown in Fig. 5, direct PEDV infection showed no apparent difference in wound healing. However, incubation with PEDV CM significantly impeded endothelial wound healing, compared with Mock CM or WIV PEDV CM incubation.

### PEDV disrupts the endothelial barrier through MMP-7 from PEDV-infected NECs.

**FIG 6:**
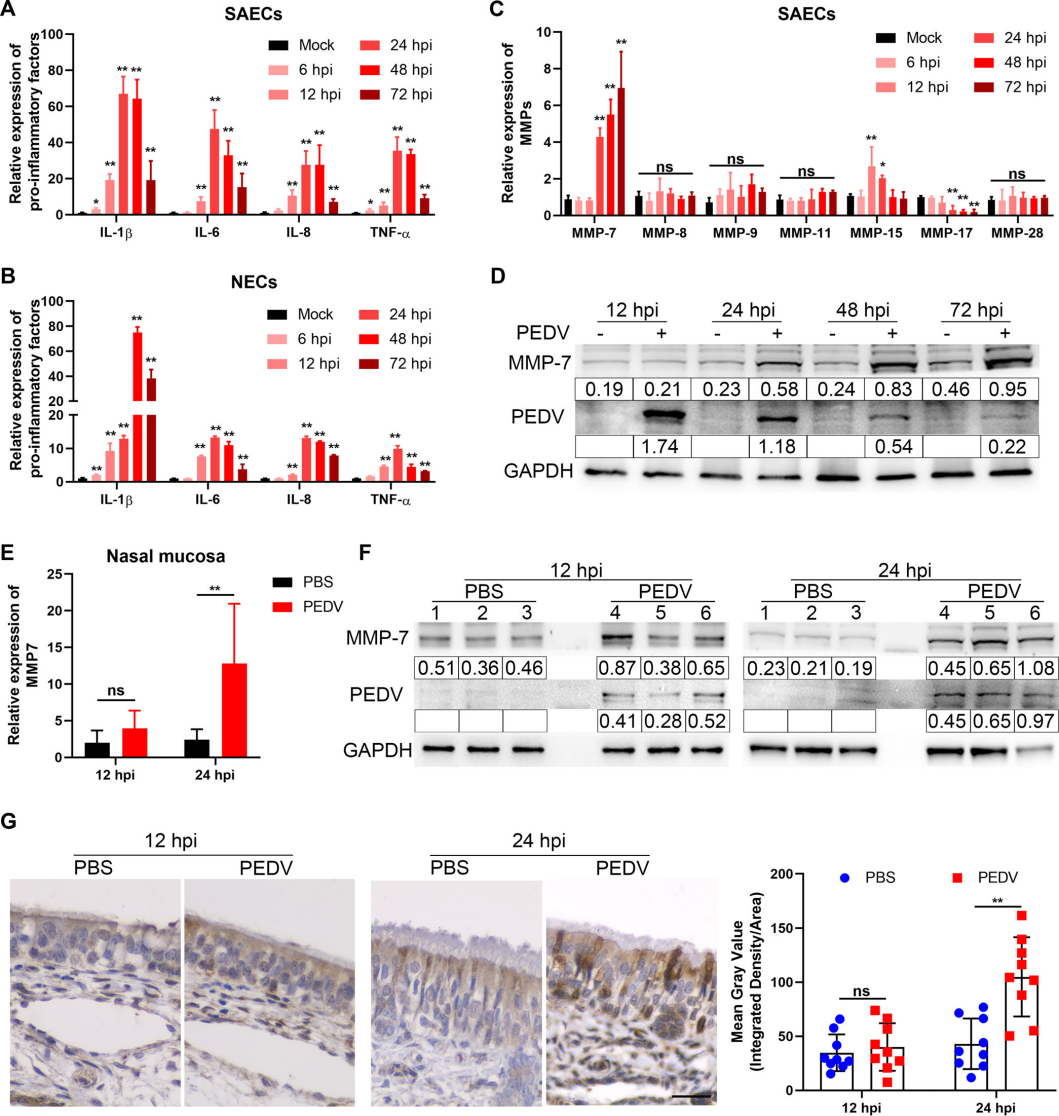
PEDV infection upregulates MMP-7 in nasal epithelium. (A and B) After PEDV infection, the levels of IL-1β, IL-6, IL-8, and tumor necrosis alpha (TNF-α) were determined in SAECs (A) and NECs (B) by RT-qPCR. (C and D) The levels of MMPs in PEDV-infected SAECs were determined by RT-qPCR (C) and Western blotting (D). (E and F) Neonatal piglets were challenged with PEDV via intranasal inoculation. The levels of MMP-7 in nasal mucosa were assessed at 12 hpi and 24 hpi by RT-qPCR (E) and Western blotting (F). (G) MMP-7 expression was visualized by IHC staining at 12 hpi and 24 hpi. The mean gray value of MMP-7 was evaluated by using ImageJ. Data are means and SD from three independent experiments. **, *P < *0.01; ns, not significant.

Proinflammatory factors (interleukins [ILs]) and matrix metalloproteinases (MMPs) participate in endothelial barrier dysfunction (20–22). Thus, expression levels of ILs and MMPs were determined in SAECs and NECs. After PEDV infection, most ILs were significantly upregulated at 12 hpi in SAECs and NECs (Fig. 6A and B). Remarkably, MMP-7 levels in SAECs showed an increasing trend after viral infection, but levels of other MMPs showed no apparent difference (Fig. 6C and D). Consistent with *in vitro* results, the expression of MMP-7 in the nasal mucosa was also increased at 24 hpi following intranasal infection with PEDV (Fig. 6E and F). Although MMP-7 expression showed no apparent difference in the early stage of PEDV intranasal infection, IHC analysis revealed PEDV intranasal infection induced high abundances of MMP-7 in the nasal epithelium at 24 hpi (Fig. 6E to G).

**FIG 7:**
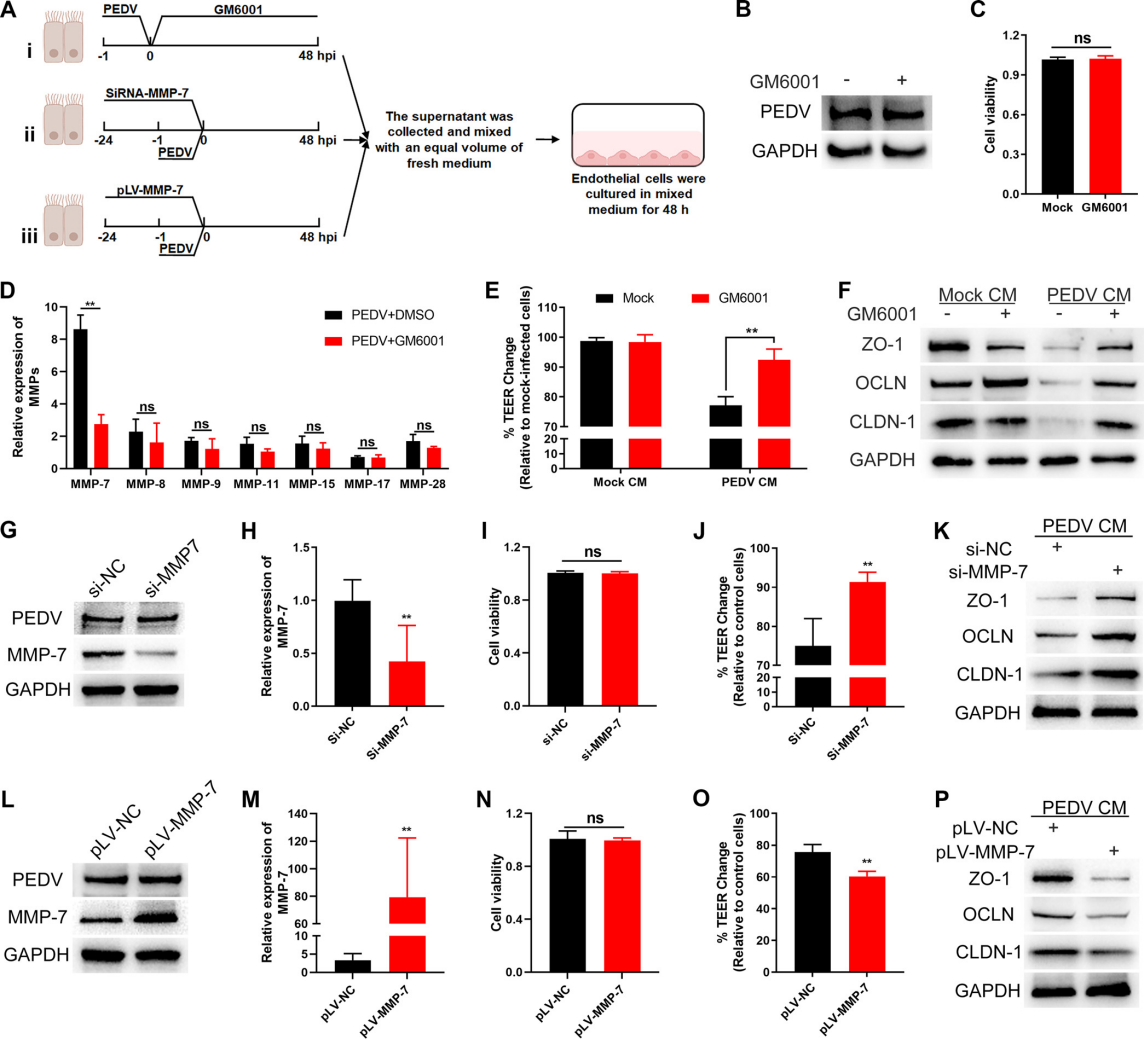
MMP-7 released by PEDV-infected NECs degrades the endothelial TJs. (A) Schematic representation of the experimental design. (Panel i) After PEDV infection, SAECs were treated with GM6001 (10 μM) for 48 h. (Panel ii) SAECs were transfected with si-MMP-7 for 24 h and then infected with PEDV for 48 h. (Panel iii) SAECs were transfected with a pLV-MMP-7 plasmid for 24 h and then infected with PEDV for 48 h. All the supernatants were collected and mixed with an equal volume of fresh medium for culturing SUVECs. (B to D) Treating SAECs with GM6001 did not affect PEDV infection (B) and cell viability (C) but impeded MMP-7 expression (D). (E and F) Confluent SUVECs were incubated with mock or PEDV CM (treatment with dimethyl sulfoxide [DMSO] or GM6001) for 48 h. The TEER was monitored (E) and the levels of ZO-1, OCLN, and CLDN-1 were determined by Western blotting (F). (G to I) Transfecting SAECs with si-MMP-7 limited MMP-7 expression (G and H) but did not affect PEDV infection (G) and cell viability (I). (J and K) Confluent SUVECs were incubated with PEDV CM (transfection with si-NC or si-MMP-7) for 48 h to determine the TEER (J) and the expression levels of ZO-1, OCLN, and CLDN-1 (K). (L to N) Transfecting SAECs with pLV-MMP-7 enhanced MMP-7 expression (L and M) but did not affect PEDV infection (L) and cell viability (N). (O and P) After SUVECs incubating with PEDV CM (transfection with pLV-NC or pLV-MMP-7), the TEER (O) and the levels of ZO-1, OCLN, and CLDN-1 (P) were evaluated at 48 h. Data are means and SD from three independent experiments. **, *P < *0.01; ns, not significant.

To further investigate the role of MMP-7 in endothelial dysfunction, SAECs were treated with the MMP inhibitor GM6001 after PEDV infection (Fig. 7A, panel i). Treatment with GM6001 had no impact on viral replication and cell viability (Fig. 7B and C) but did attenuate MMP-7 expression (Fig. 7D). After 48 h inhibitor treatment, the supernatants were mixed with an equal volume of fresh medium and used to culture the SUVECs. As shown in Fig. 7E and F, inhibiting MMP activity prevented disruption of endothelial integrity and degradation of the endothelial TJs. Furthermore, small interfering RNA (siRNA) against MMP-7 was transfected into SAECs (Fig. 7A, panel ii). The transfection with siRNA-MMP-7 (si-MMP-7) significantly reduced the expression of MMP-7 in PEDV-infected SAECs but did not affect PEDV infection or cell viability (Fig. 7G to I). Compared with that in control cells, MMP-7 silencing inhibited TEER reduction and TJ degradation in SUVECs (Fig. 7J and K). In parallel, overexpression of secreted MMP-7 by transfecting the cells with recombinant plasmid pGMLV-C-MMP-7 (pLV-MMP-7) also did not affect PEDV infection or cell viability (Fig. 7L to N). Conditioned medium transfected with pLV-MMP-7 more severely affected the disruption of endothelial integrity and the degradation of the endothelial TJs (Fig. 7O and P). These results demonstrate that MMP-7 released by PEDV-infected SAECs plays a crucial role in endothelial barrier dysfunction.

### Molecules released by PEDV-infected NECs enhance PBMC and erythrocyte adhesion to ECs and promote PBMC transendothelial migration.

**FIG 8:**
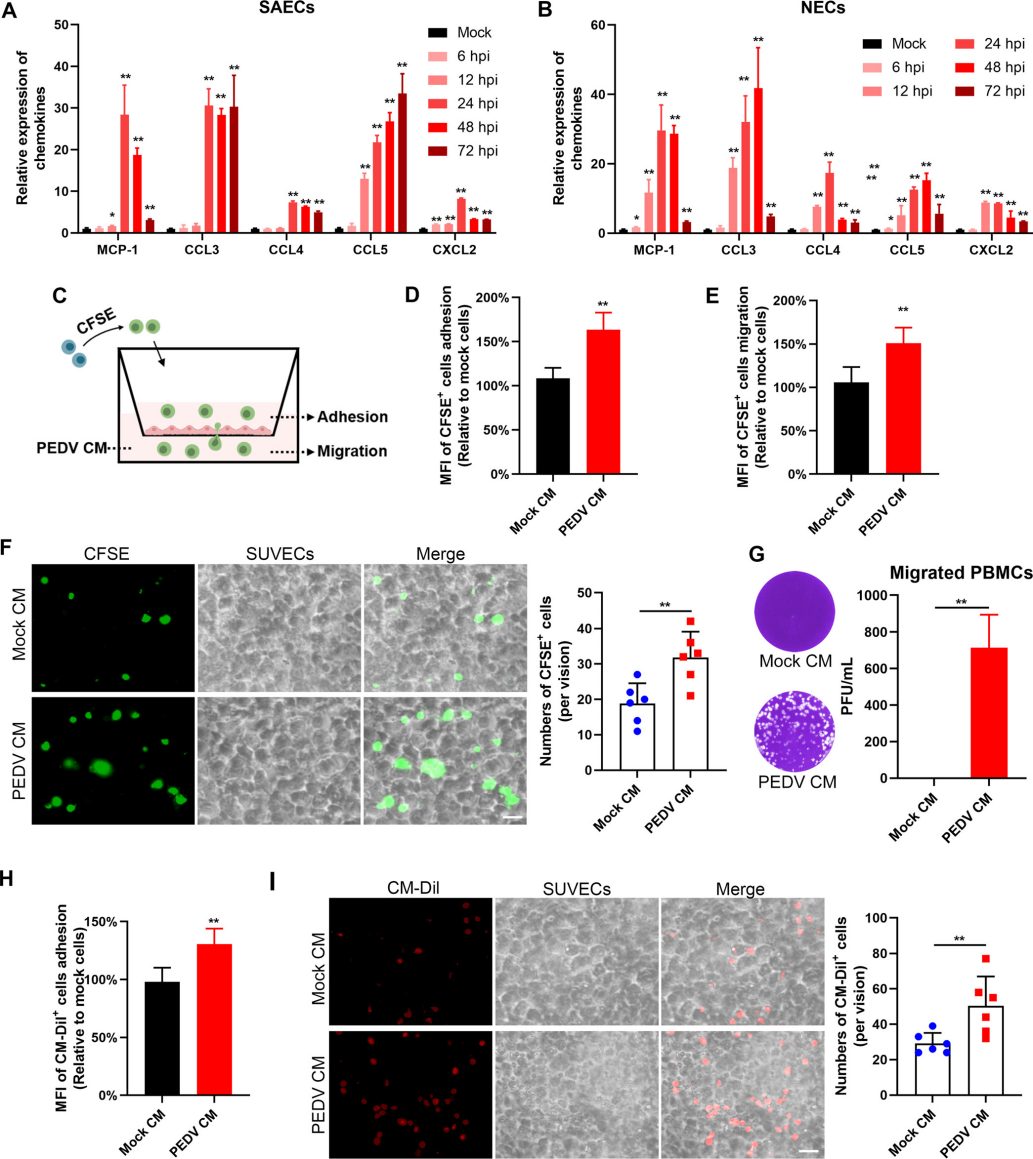
Effect of PEDV infection on adhesion of PBMCs and erythrocytes to ECs and transendothelial migration of PBMCs. (A and B) The levels of chemokines in PEDV-infected SAECs (A) and NECs (B) were determined by RT-qPCR. (C) Schematic representation of the experimental design. Confluent SUVECs were incubated with mock or PEDV CM for 24 h. CFSE-labeled PBMCs were apically added into Transwell inserts and allowed to adhere for 1 h or migrate for 16 h. (D and E) The MFIs of adherent CFSE^+^ cells (D) and migrated CFSE^+^ cells (E) were quantified. (F) CFSE-labeled PBMCs were added to SUVECs and allowed to adhere for 1 h. Adherent CFSE^+^ cells were observed with a fluorescence microscope. (G) Migrated PBMCs were collected from the lower chamber. The viral titer in migrated PBMCs was detected by plaque assay. (H and I) CM-Dil-labeled erythrocytes were added into confluent SUVECs and allowed to adhere for 1 h. Adherent CM-Dil^+^ cells were measured by MFI (H) and observed with a fluorescence microscope (I). Data are means and SD from three independent experiments. **, *P < *0.01.

The innate immune response against viral infection may also involve the production of chemokines responsible for the recruitment of immune cells to the site of infection. Hence, the mRNA expressions of several chemokines in PEDV-infected NECs and SAECs were quantified. Compared with those in mock-infected SAECs, the levels of MCP-1, CCL3, CCL4, CCL5, and CXCL2 began to be significantly upregulated at 24 hpi in PEDV-infected SAECs (Fig. 8A). Consistent with the results for PEDV-infected SAECs, the chemokines were significantly upregulated in PEDV-infected NECs (Fig. 8B). To determine whether PEDV infection contributes to the recruitment of innate immune cells, a coculture model of ECs and PBMCs was established (Fig. 8C). After incubation of SUVECs with PEDV CM for 24 h, the adhesion of PBMCs to ECs and the transendothelial migration of PBMCs were significantly enhanced by measuring the ratio of the mean fluorescence intensity (MFI) of adherent and migrated cells (Fig. 8D and E). Fluorescence observation revealed that more PBMCs adhered to ECs after incubation with PEDV CM (Fig. 8F). In parallel, PEDV was detected in migrated PBMCs and maintained viral infectivity (Fig. 8G). Given that interactions between erythrocytes and endothelium are essential in inflammation and viral infection (23), we further investigated the effect of PEDV infection on the adhesion of ECs to erythrocytes. Incubating ECs with PEDV CM caused more erythrocytes to adhere than incubation with mock CM (Fig. 8H and I).

### Involvement of ICAM-1 mediated by NF-κB signaling activation in PBMC transendothelial migration.

**FIG 9:**
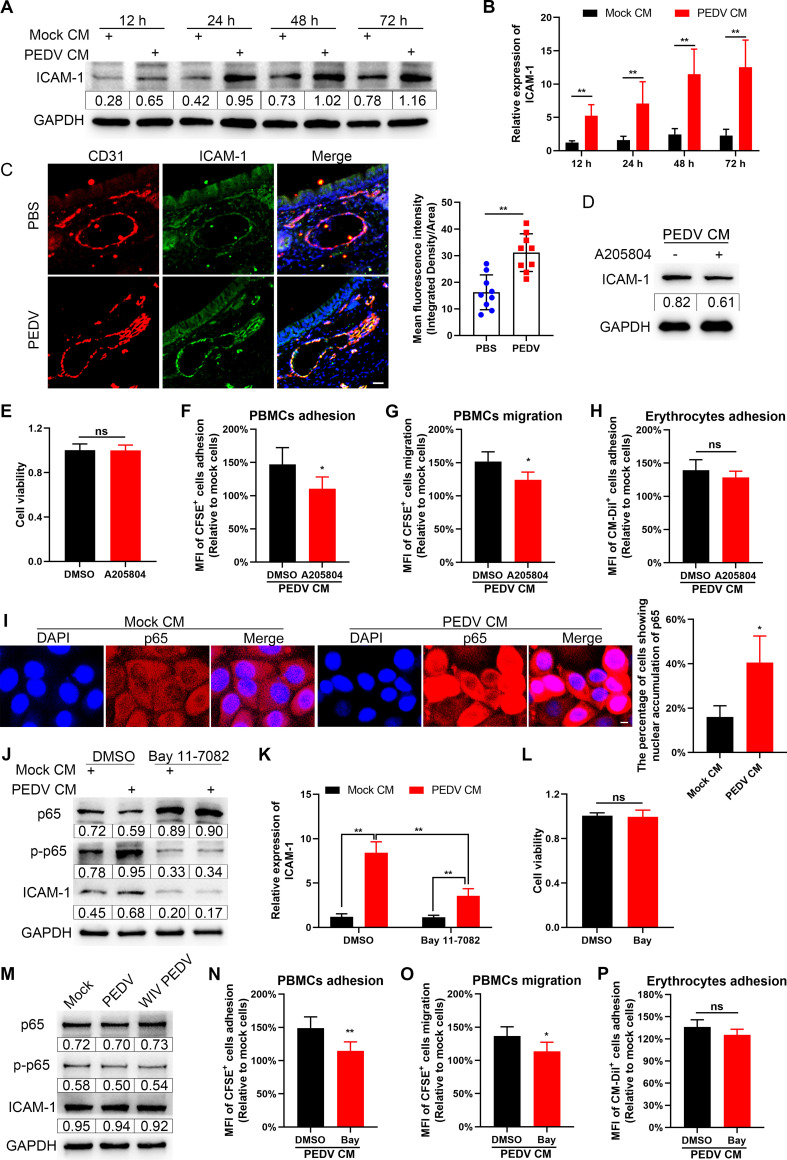
Involvement of ICAM-1 mediated by NF-κB signaling in PBMCs migration. (A and B) After incubation of SAECs with mock or PEDV CM, ICAM-1 expression levels were determined by Western blotting (A) and RT-qPCR (B). (C) Neonatal piglets were challenged with PEDV via intranasal inoculation. The expression levels of ICAM-1 in nasal microvessels were visualized by IFA staining. Blue, DAPI; red, CD31; green, ICAM-1. Bars, 25 μm. The MFI of ICAM-1 was evaluated by using ImageJ. (D to H). Confluent SUVECs were treated with A205804 (100 nM) in the presence of PEDV CM for 24 h. CFSE-labeled PBMCs and CM-Dil-labeled erythrocytes were apically added into Transwell inserts and allowed to adhere for 1 h or migrate for 16 h. Treating SAECs with GM6001 limited ICAM-1 expression (D) but did not affect cell viability (E). The MFIs of adherent CFSE^+^ PBMCs (F), migrated CFSE^+^ PBMCs (G), and adherent CM-Dil^+^ erythrocytes (H) were quantified using a fluorometer. (I) After incubation of SUVECs with mock or PEDV CM for 24 h, the nuclear translocations of p65 were observed by IFA. Blue, DAPI; red, p65. Bars, 5 μm. Nuclear accumulation of p65 was evaluated in 100 cells in three independent experiments. (J to L) SUVECs were pretreated with DMSO or Bay 11-7082 (10 μM) for 2 h and then incubated with mock or PEDV CM for 24 h. The levels of p65, p-p65, and ICAM-1 were assessed by Western blotting (J) and RT-qPCR (K). The viability of SUVECs was evaluated with a CCK-8 kit (L). (M) SUVECs were directly inoculated with PEDV or WIV PEDV (MOI, 1). The expression levels of p65, p-p65, and ICAM-1 were determined at 24 hpi by Western blotting. (N to P) SUVECs were pretreated with DMSO or Bay 11-7082 for 2 h and incubated with PEDV CM for 24 h. CFSE-labeled PBMCs and CM-Dil-labeled erythrocytes were apically added to the SUVECs and allowed to adhere for 1 h or migrate for 16 h. The MFIs of adherent CFSE+ PBMCs (N), migrated CFSE+ PBMCs (O), and adherent CM-Dil+ erythrocytes (P) were measured using a fluorometer. Data are means and SD from three independent experiments. *, *P < *0.05; **, *P < *0.01; ns, not significant.

Intercellular adhesion molecule-1 (ICAM-1) is a critical regulator of high-affinity interactions between ECs and innate immune cells (24, 25). Thus, ICAM-1 expression was detected after incubation of SUVECs with PEDV CM. As shown in Fig. 9A and B, incubation with PEDV CM increased ICAM-1 expression. Similar to the *in vitro* results, PEDV intranasal infection induced ICAM-1 upregulation in nasal microvessels by IFA observation (Fig. 9C). To further investigate the role of ICAM-1 in endothelial adhesion, the SUVECs were treated with an ICAM-1 inhibitor A205804 during PEDV CM incubation. Treatment with A205804 attenuated ICAM-1 expression but did not affect the SUVECs viability (Fig. 9D and E). Inhibition of ICAM-1 expression decreased PBMC adhesion and transendothelial migration (Fig. 9F and G). However, inhibiting ICAM-1 expression did not affect the adhesion of ECs to erythrocytes (Fig. 9H).

We further investigated the mechanism by which PEDV CM induces ICAM-1 in ECs. Considering that NF-κB signaling is the central coordinating signaling that mediates innate immune responses, we evaluated the activation of NF-κB signaling in ECs. Phosphorylation and nuclear translocation of p65 are pivotal indicators of NF-κB signaling activation (26). Compared with mock CM incubation, PEDV CM incubation increased the nuclear translocation of p65 by IFA observation (Fig. 9I). Moreover, more phosphorylation of p65 in SUVECs was verified following incubation with PEDV (Fig. 9J), further verifying that the molecules released by PEDV-infected SAECs activates the NF-κB signaling. In contrast, pretreating the SUVECs with an inhibitor of NF-κB, Bay 11-7082, significantly impeded PEDV CM-induced ICAM-1 expression (Fig. 9J and K), but without affecting cell viability (Fig. 9L). However, direct inoculation with PEDV did not affect the NF-κB signaling and ICAM-1 expression in SUVECs (Fig. 9M), indicating that PEDV cannot activate ECs. Furthermore, inhibiting NF-κB signaling in ECs attenuated the adhesion and transendothelial migration of PBMCs (Fig. 9N and O) but did not affect adhesion of ECs to erythrocytes (Fig. 9P). These findings imply that ILs released by PEDV-infected NECs elevate ICAM-1 expression in ECs by activating NF-κB signaling. This activated phenotype enhances the transmigration of PBMCs across ECs.

## DISCUSSION

As a vital component of the mucosal immunity in the upper airways, the nasal mucosa forms a continuous barrier against exogenous pathogens in the nasal epithelium. In contrast, the epithelium is the first site of exposure to invading pathogens and is one of the main routes of viral entry (1–3, 27, 28). In many airborne viral diseases, penetrating the nasal epithelium is the first step of infection. Following intranasal inoculation, PEDV appeared in various tissues and blood cells. However, how PEDV crosses the endothelial barrier into the bloodstream is unclear. Therefore, this study aimed to elucidate the role of the endothelial barrier in this process.

The endothelium forms a semipermeable barrier that separates the circulating blood from the peripheral tissue. Endothelial dysfunction results in serious pathological consequences, such as viremia, sepsis, and tissue damage (29, 30). We found that intranasal infection with PEDV elevated PLVAP expression, suggesting that the viral infection disrupted the integrity of nasal subepithelial microvessels. However, PEDV could not directly interact with the ECs, replicate in these cells, and compromise the endothelial barrier, possibly because ECs lack receptors that bind PEDV. PEDV mainly infects villous epithelial cells or enterocytes in the small intestine. Porcine aminopeptidase N (APN) and sialic acid have both been identified as functional receptors for PEDV, but these findings are controversial (31, 32). Our previous study has demonstrated that the susceptibility of newborn piglets to PEDV is related to transferrin receptor expression, indicating that the transferrin receptor might be an entry receptor of PEDV (33). In addition to direct viral damage, excessive mediators released from neighboring cells also impair endothelial integrity. For instance, endothelial dysfunction induced by HIV-1 infection potentially depends on molecules released into the microenvironment by HIV-1-infected cells rather than the virus itself (34, 35). Moreover, several flaviviruses, including Zika virus and West Nile virus, selectively disrupt the endothelial barrier by secreting viral proteins (36). In SARS-CoV-2 infection, persistent endothelial dysfunction results from direct effects of viral infection and indirect consequences of excessive ILs (37, 38). In this study, PEDV infection compromised the endothelial barrier through molecules released from PEDV-infected NECs, resulting in the degradation of TJs, including ZO-1, OCLN, and CLDN-1. Interestingly, molecules released from PEDV-infected NECs also play an important role in endothelial barrier repair.

Various endogenous (e.g., ILs, hormones, and proteinases) and exogenous (e.g., drugs, toxins, and invading pathogens) stimuli impinge on the endothelial barrier to affect its integrity and permeability (39, 40). However, overwhelming or persistent stimulation can disrupt the endothelial barrier by degrading endothelial junctions. PEDV infection elevated MMP-7 expression in the nasal epithelium. As a member of the metalloproteinase family, MMP-7 plays an indispensable role in ECM remolding, proteolytic activity, and cell migration (20). Moreover, we discovered that the released MMP-7 could degrade endothelial TJs, including ZO-1, OCLN, and CLDN-1. In summary, PEDV indirectly disrupts the endothelial barrier via MMP-7 released from PEDV-infected NECs.

In addition to the barrier function, ECs orchestrate local innate immune responses and leukocyte recruitment. ECs maintain tight control over leukocyte migration from the blood to tissues. When viruses provoke the excessive release of ILs and chemokines at the entry site, ECs capture the released cytokines in the virus-induced microenvironment and present them on their luminal surfaces to recruit immune cells (41). ILs and chemokines in the virus-induced microenvironment also stimulate the upregulation of adhesion molecules in ECs to regulate leukocyte rolling and adhesive interactions with endothelium (24). During HIV-1 infection, gamma interferon (IFN-γ)-mediated enhancement of ICAM-1 expression on the surface of ECs promotes the adhesion of HIV-1^+^ T cells to ECs and transendothelial migration (42, 43). Similarly, PEDV infection enhanced the release of ILs and chemokines from NECs. The released cytokines promoted the adhesion of PBMCs to ECs and PBMC transendothelial migration by upregulating ICAM-1 expression on the surface of ECs. As central signaling that regulates inflammatory responses, NF-κB signaling has a crucial role in regulating endothelial surface adhesion molecules and mediating leukocyte adhesion (25). In our study, NF-κB signaling activation was associated with the upregulation of ICAM-1 and the mobilization of ECs for the adhesion to PBMCs. The cytokines released from PEDV-infected NECs also enhanced the adhesion of erythrocytes to ECs, but the detailed mechanism has not been defined. Taken together, our results show that cytokines released from PEDV-infected NECs enhance ICAM-1 expression in ECs through NF-κB signaling activation to promote PBMC migration.

Innate immune cells are recruited by a host of ILs and chemokines for effective immune response and viral clearance. However, many viruses have evolved strategies to exploit innate immune cells to promote their dissemination. A valuable example is the Nipah virus, which attaches to lymphocytes that do not support its replication but facilitate its trans-infection of ECs (44). In addition, a multitude of emerging viruses, including dengue virus, measles virus, and Zika virus, may exploit innate immune cells to migrate across the endothelial barrier, disseminate in the body, and cause disease (45–47). Our study showed that PEDV was present in migrated PBMCs *in vitro* and PBMCs in blood after PEDV intranasal inoculation, indicating that PEDV exploits PBMCs to spread through blood.

**FIG 10:**
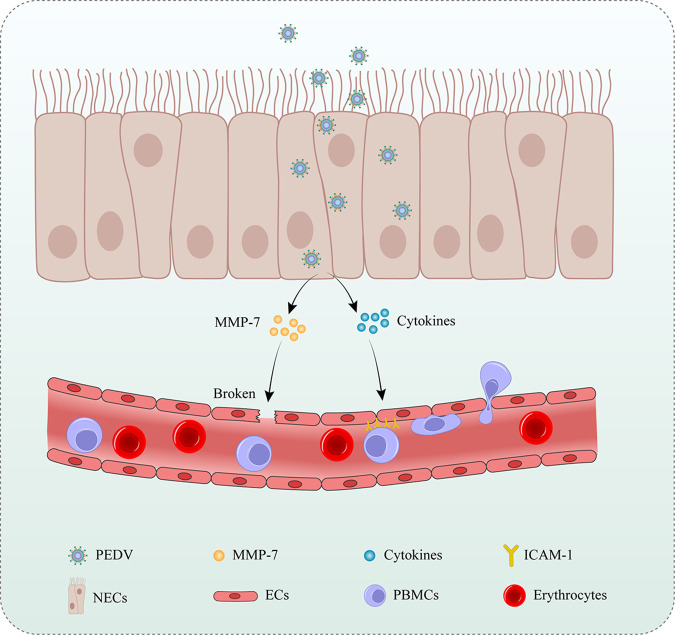
Schematic of the proposed mechanism for PEDV disrupting the endothelial barrier. PEDV infection of NECs induces the upregulation of MMP-7 and cytokines. The released MMP-7 disrupts the endothelial integrity by degrading the endothelial TJs. Moreover, the released cytokines activate ECs upregulating ICAM-1 to facilitate PBMC migration.

In conclusion, our study demonstrates an underlying mechanism by which PEDV disrupts the endothelial barrier through indirect effects (Fig. 10). PEDV exhibits transient tropism in NECs, upregulating MMP-7, ILs, and chemokines. The released MMP-7 disrupts the endothelial barrier by degrading the endothelial TJs. Meanwhile, the released ILs and chemokines activated ECs to promote PBMC migration. PEDV could further exploits PBMCs to favor its dissemination. Our findings address the role of PEDV infection in endothelial dysfunction and provide novel insights into the pathogenesis of disseminated viral disease with the same characteristics, which can be valuable for developing effective strategies for preventing emerging viruses.

## MATERIALS AND METHODS

### Ethics statement.

All animal challenges were approved by the Institutional Animal Care and Use Committee of Nanjing Agricultural University (SYXK-2017-0027) and followed the National Institutes of Health guidelines for animal experiments.

### Cells and viruses.

Porcine NECs were isolated and generated as previously described (5). The acquired NECs were seeded in 12-well inserts of Transwells (3-μm pore size) coated with collagen type IV at a concentration of 2 × 10^5^ cells per insert. Porcine PBMCs were isolated by density centrifugation using a porcine peripheral blood lymphocyte separation kit (Solarbio) and cultured in RPMI 1640 medium with 10% fetal bovine serum (FBS) at 37°C with 5% CO_2_. Porcine erythrocytes were isolated from whole blood as previously described (14) and stored in red cell storage solution (Solarbio) at 4°C. SUVECs and Vero E6 cells were cultured in Dulbecco’s modified Eagle’s medium (DMEM), and SAECs were cultured in RPMI 1640 medium at 37°C with 5% CO_2_. All cells were supplemented with 10% FBS and penicillin/streptomycin (100 μg/mL). PEDV strain Zhejiang08 was isolated from piglets exhibiting serious diarrhea in 2012 (4) and was grown and titrated in Vero E6 cells.

### Intranasal inoculation with PEDV.

Neonatal piglets of similar weights were purchased from a herd that had a high health status and had been confirmed as seronegative and pathogen negative for the following viruses: PEDV, transmissible gastroenteritis virus, porcine reproductive and respiratory syndrome virus, classical swine fever virus, porcine circovirus type 2, and porcine respiratory coronavirus. The piglets were randomly allocated to a control group and the PEDV infection group (*n* = 6 per group). Each group was housed in a separate room at constant humidity and temperature. The piglets in the PEDV infection group were challenged with 1 mL PEDV (10^7^ PFU/mL) via nasal inoculation. In contrast, neonatal piglets in the control group were treated with an equal volume of sterile phosphate-buffered saline (PBS). The control group was treated with an equal volume of sterile PBS. Nasal inoculation was performed using a nasal spray device, as previously described (4). The piglets (3 per group) were euthanized to collect samples at 12 hpi and 24 hpi.

### PEDV infection.

After a confluent monolayer had been grown, NECs, SAECs, and SUVECs were inoculated with PEDV (MOI, 1) for 1 h at 37°C. After the viral inoculum was discarded, the cells were washed and cultured in corresponding maintenance media (containing 2% FBS) for further incubation at 37°C.

### TEER and permeability assay.

SUVECs were grown on 24-well Transwell inserts (3-μm pore size) to confluence. After SUVECs incubating with mock or PEDV CM, the TEER was monitored using a Millicell ERS ohmmeter (Millipore). For transendothelial permeability assays, after SUVECs had been incubated with mock or PEDV CM, dextran-FITC (fluorescein isothiocyanate) was added to the SUVECs at a concentration of 0.1 μg/mL for 30 min. The medium from the lower chamber was sampled for fluorescence measurements and compared with control monolayers. Fluorescence was quantified using a fluorometer (excitation wavelength [λ_ex_] = 492 nm; emission wavelength [λ_em_] = 520 nm). All values were expressed as percentages relative to values in the control cells.

### Scratch assay.

SUVECs were seeded into six-well plates to more than 95% confluence. The cells were wounded with a 200-μL pipette tip and then gently washed to remove detached cells. After incubation with mock or PEDV CM, the cells were observed each 6 h until wound healing.

### RNA interference and overexpression of MMP-7.

For RNA interference, SAECs were transfected with 100 nM MMP-7-specific or scrambled control siRNA duplexes (Table 1) using the X-tremeGENE HP DNA transfection reagent (Roche) according to the manufacturer’s instructions. After 24 h of transfection, the cells were infected with PEDV (MOI, 1) for subsequent analysis.

**TABLE 1:** siRNA sequences used for RNA interference

siRNA	Orientation	Sequence (5′–3′)
Porcine MMP-7	Sense	GUGGCAGCAUAGGCAUUAATT
	Antisense	UUAAUGCCUAUGCUGCCACTT
Negative control	Sense	UUCUCCGAACGUGUCACGUTT
	Antisense	ACGUGACACGUUCGGAGAATT

For MMP-7 overexpression, the MMP-7 gene (NM_001348795.1) was amplified from the cDNA of porcine nasal epithelium using the corresponding primers (Table 2) and subcloned into the pGMLV-C-flag vector at the XhoI and EcoRI I sites. Nucleotide sequences of MMP-7-expressing plasmid were verified to ensure that the correct clone was used in the study. The MMP-7-expressing and control plasmids were transfected into SAECs using the X-tremeGENE HP DNA transfection reagent. After 24 h of transfection, the cells were infected with PEDV at an MOI of 1 for further analysis.

**TABLE 2:** PCR primers used in this study

Gene	Direction	Sequence (5′–3′)
Porcine MMP-7 (for plasmid construction)	Forward	GCTACCGGACTCAGATCTCGAGGCCACCATGCAACTGGCTGTACTGTGTG
	Reverse	CTCACCATACCACTACCGAATTCATTCTTTTCTGGGTTACTTCTCTTTCC
PEDV (N)	Forward	CACCTCCTGCTTCACGTACA
	Reverse	AGCTCCACGACCCTGGTTAT
Porcine GAPDH	Forward	TCATCATCTCTGCCCCTTCT
	Reverse	GTCATGAGTCCCTCCACGAT
Porcine PLVAP	Forward	TCCTGTGCCAACTACTGAGC
	Reverse	AATACTTCCTCTGCCTGGCT
Porcine IL-1β	Forward	GAAAGCCATACCCAGAGGTC
	Reverse	GCACTAATCTAGGGAAGACAGC
Porcine IL-6	Forward	TGGGTTCAATCAGGAGACCT
	Reverse	CAGCCTCGACATTTCCCTTA
Porcine IL-8	Forward	GAAGAGAACTGAGAAGCAACAACA
	Reverse	TTGTGTTGGCATCTTTACTGAGA
Porcine TNF-α	Forward	CCCCCAGAAGGAAGAGTTTC
	Reverse	CGGGCTTATCTGAGGTTTGA
Porcine MMP-7	Forward	GGAACAGGCTCAGGGCTATC
	Reverse	CTGGTACTCCACATCTGGGC
Porcine MMP-8	Forward	CCTACTGGACCAACCACACC
	Reverse	CAGCCTGTATGCCATTTCGC
Porcine MMP-9	Forward	ACTTCGGAAACGCAAAAGGC
	Reverse	TACCGTCCCGAGTGAAGAGT
Porcine MMP-11	Forward	CTTCTTCCCCAAGACCCACC
	Reverse	GATACCCCTGCGGTCATCTG
Porcine MMP-15	Forward	GGACATGGTACTGTAGAGCCG
	Reverse	GCTTACAAGGGAGGCACACT
Porcine MMP-17	Forward	TCTGAGAGCTTGGACGGAGA
	Reverse	TCATCGAGTGTCATAGCGCC
Porcine MMP-28	Forward	ACCTGTACGACAGGCAACAG
	Reverse	CCCACCTTTCCTGTAGTGGG
Porcine MCP-1	Forward	GCGGCTGATGAGCTACAGAAG
	Reverse	CCGCGATGGTCTTGAAGATC
Porcine CCL3	Forward	GTCGCCGTGGCTGCTCTC
	Reverse	CGGCTACGAATTTGCGAGGAAG
Porcine CCL4	Forward	GCAAGACCATGAAGCTCTGC
	Reverse	AAGCTTCCGCACGGTGTATG
Porcine CCL5	Forward	ATCAGCCTCCCCATATGCCT
	Reverse	CCGCACCCATTTCTTCTCTG
Porcine CXCL2	Forward	CCGTGCAAGGAATTCACCTC
	Reverse	TGCGGGGTTGAGACAAACTT
Porcine ICAM-1	Forward	GAGCTGTTCAGGCAGTCAGT
	Reverse	GTTCACAGAAACGGGTGTGC

### Adhesion assay.

PBMCs and erythrocytes were stained with carboxyfluorescein succinimidyl ester (CFSE) and chloromethyl-benzamidodialkylcarbocyanine (CM-Dil), respectively. SUVECs were seeded on coverslips placed in 24-well plates until confluence was reached. After incubation of SUVECs with mock or PEDV CM for 24 h, the confluent SUVECs were cocultured with CFSE-labeled PBMCs or CM-Dil-labeled erythrocytes (1 × 10^5^ cells/well) for 1 h. The nonadherent cells were removed by washing three times with PBS. The adherent cells were fixed with 4% paraformaldehyde for analysis using fluorescence microscopy or lysed with radioimmunoprecipitation assay (RIPA) buffer for quantifying the MFI.

### Transmigration assay.

SUVECs were seeded on polycarbonate microporous membranes of 12-well Transwell chambers and incubated to confluent monolayers. The cells were incubated with mock or PEDV CM for 24 h. CFSE-labeled PBMCs (1 × 10^5^ cells/well) were added to the top chamber and allowed to migrate for 16 h at 37°C. The migrated cells were collected from the lower chamber. CFSE fluorescence (λ_ex_ = 494 nm; λ_em_ = 521 nm) and CM-Dil fluorescence (λ_ex_ = 553 nm; λ_em_ = 571 nm) were quantified using a fluorometer.

### IHC and IFA.

Nasal tissues were fixated with 4% paraformaldehyde and embedded in paraffin. Tissue sections were subsequently incubated with anti-PEDV-N antibody (Median Diagnostics) and anti-MMP-7 antibody (Abcam) to examine the levels of PEDV and MMP-7 by IHC staining. For immunofluorescent assay (IFA), tissue sections and cells were incubated with primary antibodies overnight at 4°C and then stained with the corresponding secondary antibodies, and nuclei were stained with DAPI (4′,6-diamidino-2-phenylindole). Tissue sections and cells were observed using a Zeiss LSM710 confocal microscope.

### RT-qPCR.

Total RNAs from cells and tissues were extracted with RNAiso Plus (TaKaRa). Reverse transcriptions were completed using a HiScript III 1st Strand cDNA synthesis kit (Vazyme). Reverse transcription-quantitative PCR (RT-qPCR) was performed by using a TB Green qPCR kit (TaKaRa) in the Applied Biosystems 7500 fast real-time PCR system (Life Technologies). All primers are shown in Table 2. The GAPDH (glyceraldehyde-3-phosphate dehydrogenase) gene was quantified as the reference gene.

### Western blotting.

Samples were lysed using RIPA lysis buffer, and protein concentrations were determined using a bicinchoninic acid (BCA) assay. Proteins were separated by SDS-PAGE and transferred onto polyvinylidene difluoride membranes. After blocking with 5% skim milk, the membranes were explored with specific primary antibodies. Mouse monoclonal antibody targeting PEDV N protein was purchased from Medgene Labs. Antibodies against PLVAP, ZO-1, OCLN, CLDN-1, and ICAM-1 were obtained from Proteintech. Antibody against MMP-7 was from Abcam. NF-κB signaling was evaluated using anti-p65 and anti-phospho-p65 antibodies from Cell Signaling Technology. GAPDH was used as the internal control to evaluate the protein amounts by using ImageJ.

### Flow-cytometric analysis.

After intranasal inoculation with PEDV, PBMCs and erythrocytes were isolated from neonatal-piglet blood and then resuspended in fixation/permeabilization solution (BD Cytofix/Cytoperm kit; BD Pharmingen). After staining with anti-PEDV-N-FITC antibody (Median Diagnostics), the cells were analyzed by flow-cytometric (BD FACSCalibur) analysis.

### Plaque assay.

Vero E6 cell monolayers were inoculated with virus stock (1 mL of serial 10-fold dilutions) for 1 h at 37°C with 5% CO_2_. Then, the cells were overlaid with 0.8% low-melting-point agarose in DMEM containing 2% FBS and incubated at 37°C for 3 days. The cells were stained with crystal violet to observe plaques.

### Statistical analysis.

Results were determined as means and standard deviations (SD) by using SPSS 15.0 software (SPSS Inc.). RT-qPCR and Western blotting results were analyzed using analysis of variance or *t* tests. For all analyses, a *P* value of <0.05 was considered statistically significant and a *P* value of <0.01 was considered highly statistically significant.
